# Measures to support informal care for the older adults in Kazakhstan: a review of the current status

**DOI:** 10.3389/fpubh.2023.1247684

**Published:** 2023-08-21

**Authors:** Aliya Zhylkybekova, Andrey Turlayev, Andrej M. Grjibovski, Gulbakit K. Koshmaganbetova

**Affiliations:** ^1^Department of Evidence-Based Medicine and Scientific Management, West Kazakhstan Marat Ospanov Medical University, Aktobe, Kazakhstan; ^2^Department of Law, Buketov Karaganda University, Karaganda, Kazakhstan; ^3^Central Scientific Research Laboratory, Northern State Medical University, Arkhangelsk, Russia; ^4^Department of Epidemiology and Modern Vaccination Technologies, I.M. Sechenov First Moscow State Medical University, Moscow, Russia; ^5^Department of Biology, Ecology and Biotechnology, Northern (Arctic) Federal University, Arkhangelsk, Russia; ^6^Department of Health Policy and Management, Al-Farabi Kazakh National University, Almaty, Kazakhstan

**Keywords:** Kazakhstan, caregiver, older adults, social support, medical support, legislation

## Abstract

The demand for informal caregivers to support the older adults has grown worldwide in recent decades. However, informal caregivers themselves require support. This article aims to examine existing support measures for caregivers of the older adults in the Republic of Kazakhstan. Relevant articles and grey literature were identified through manual searches on Google and Google Scholar, as well as electronic searches using indexed databases like PubMed, Web of Science, and Scopus. Moreover, the reference lists of identified sources and government ministry websites were meticulously scrutinized. This review highlights the scarcity of research on caregiver support measures in Kazakhstan, supported by the lack of peer-reviewed articles on this subject. A comprehensive analysis of the literature shows that in Kazakhstan’s legislative framework, “caregivers” exclusively refers to individuals providing care for a first-degree disability. The responsibility of caring for older adults parents lies with able-bodied children. However, there is a lack of registration and assessment procedures to evaluate the burden and quality of life of caregivers. As a result, the medical and social support provided to caregivers is standardized, failing to adequately address their unique needs and requirements. The analysis of current support measures for informal caregivers highlights the need to develop support mechanisms and recognize individuals providing informal care as key figures in the long-term care system.

## 1. Introduction

The global population of older individuals is continuously growing in both absolute numbers and relative proportions. As reported by the World Health Organization (WHO), in 2010, there were 524 million people aged 65 and above, and this number is projected to reach 1.5 billion by 2050 (1). Similarly, the age composition of the population in Kazakhstan is also experiencing a transformation in line with these global patterns. By 2050, the population of individuals aged 65 and older is expected to double from the 2019 figures, increasing from 1.4 million to 3.4 million. Consequently, their share of the total population of the country will rise from 7.5% in 2019 to over 14% by 2050 (2). The United Nations (UN) Population Fund (UNFPA) notes that the demographic situation in the northeastern region of the country and certain parts of central Kazakhstan bears resemblance to that of European countries (3).

Recent statistics indicate that approximately one in every five older adults individuals surveyed in Kazakhstan requires some form of assistance. The need for assistance is particularly prevalent among those aged 60–69 years (22%) and those above 70 years (31%). When faced with the need for physical support, 69% of individuals over the age of 65 seek assistance from their children, while a mere 0.8% seek help from social services (4). This trend may be attributed to socio-cultural factors and the underdeveloped nature of the formal care system (5, 6). The growing number of older individuals with specific needs subsequently amplifies the demand for both formal and informal caregivers.

Informal care emerges as a viable substitute for formal long-term care in the context of older adults individuals. By relying on informal caregivers, older adults can maintain their residence in familiar surroundings, thereby mitigating the strain on healthcare and social welfare systems, while concurrently alleviating the burden on the state budget (1). However, it is important to acknowledge that assuming caregiving responsibilities can yield both advantageous and detrimental effects on the mental and physical well-being of informal caregivers (2). Table 1 presents a comprehensive list of these effects.

**Table 1:** Positive and negative effects of care on informal caregivers.

Positive effect	Ref	Negative effect	Ref
Family solidarity	(7, 8)	Risk of cardio-vascular diseases	(9, 10)
Learning knowledge and skills	(8)	Disruption of regular sleep	(11–13)
Affection, compassion	(8)	Risk of Diabetes mellitus 2 type	(14)
Self-confidence	(8)	Anxiety symptoms	(13, 15)
Personal growth	(8)	Social isolation	(16–18)
		Financial difficulties	(19, 20)
		Decrease work performance	(21, 22)

Various nations have different approaches to providing assistance and services to caregivers responsible for the well-being of older adults individuals in need of care. These approaches are primarily influenced by factors such as the country’s income level, legislative and sociocultural characteristics, and the type of funding allocated to the long-term care system.

Primary healthcare plays a pivotal role in this system, encompassing comprehensive aspects of individuals’ physical, mental, and social well-being. Adopting such an approach enables the delivery of integrated care across individuals’ lifespans, including health promotion, disease prevention, treatment, and rehabilitation, all tailored to align with their everyday lives. Such considerations hold particular significance for informal caregivers who undertake the responsibility of caring for the older adults (3).

State support for informal care in Kazakhstan is in the developmental stage and requires substantial improvements. The strategic documents of the Republic of Kazakhstan related to health and social support lack adequate provisions for identifying and assessing caregivers’ burden and needs, as well as providing financial support and ensuring occupational health conditions for these individuals.

Previous studies conducted in Kazakhstan have primarily focused on the needs of older individuals (4–6) or assessing the competencies of caregivers in caring for critically ill patients (23). Significantly, there is a lack of official data on the current number of formal and informal caregivers operating in the Republic of Kazakhstan.

Presumably, the majority (95%) of caregivers are informal and do not receive sufficient medical and social support tailored to their specific needs. This analysis is significant in facilitating the effective development of support mechanisms for familial care of the older adults and individuals with disabilities in Kazakhstan.

## 2. Methods

### 2.1. Search strategy

**Figure 1:**
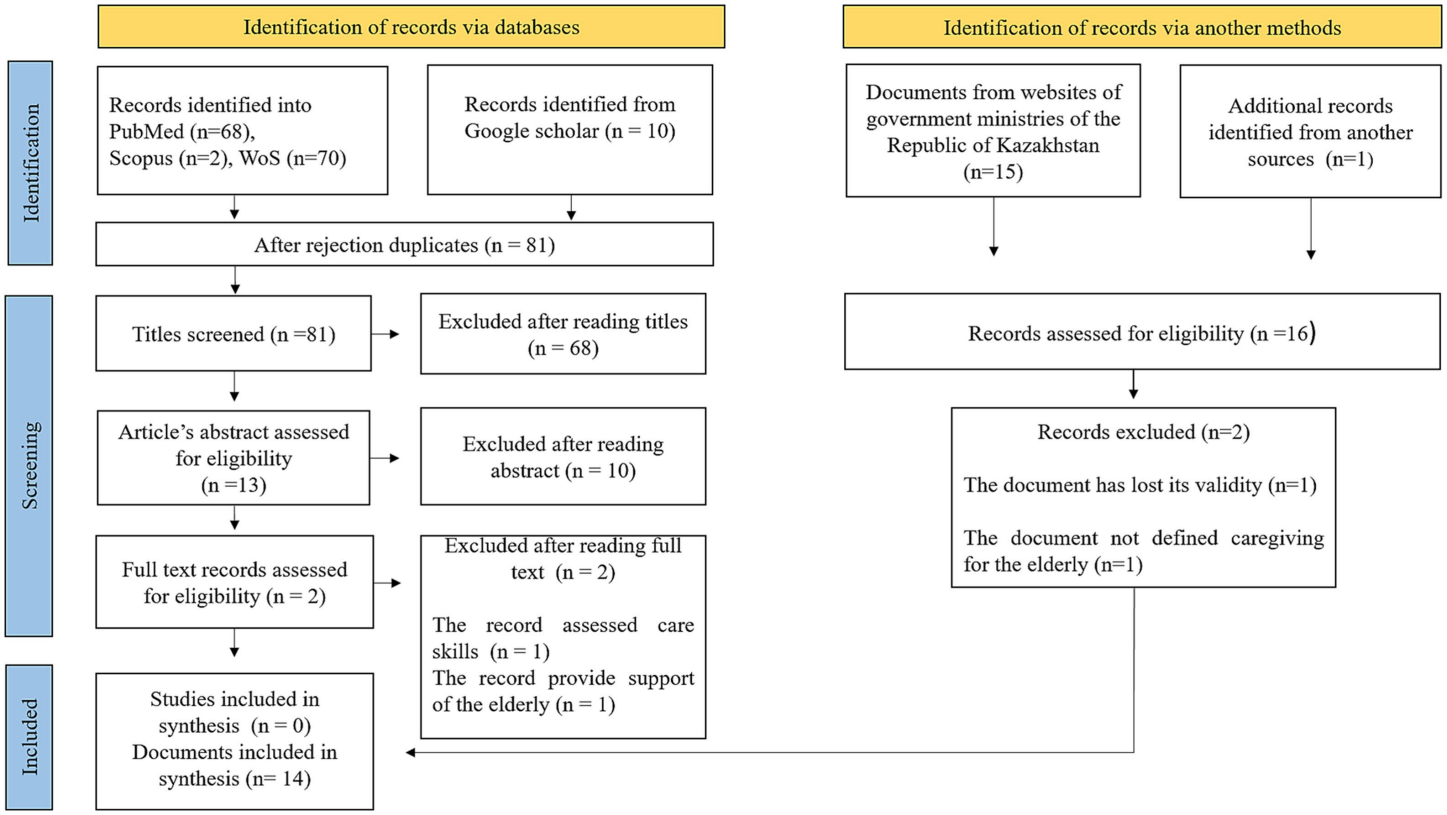
PRISMA flow diagram.

In our scoping review, relevant articles and grey literature were identified through manual searches on Google and Google Scholar, as well as electronic searches using indexed databases like PubMed, Web of Science, and Scopus. Additionally, the reference lists of all identified sources and the websites of government ministries were thoroughly reviewed. Figure 1 presents the details of the screening process, indicating that a total of 14 records out of 166 were included in our final synthesis. The websites address of government ministries is presented in Supplementary Table S1. The search was conducted using Medical Subject Headings (MeSH) and keywords, both separately and in combination, with the use of Boolean operators (AND/OR). The search strategies are presented in Supplementary Table S2. All records had to be in full text and written in English, providing comprehensive information regarding the policies supporting informal caregivers of the older adults in the Republic of Kazakhstan. The review period spanned from January 28, 2023, to June 1, 2023. We used the reference manager software program EndNote to download relevant citations and subsequently eliminated any duplicate articles. Following this, we exported the obtained data to Excel for further examination and analysis in the review process.

### 2.2. Study selection and data extraction

Two authors (AZ and AT) conducted a rigorous analysis of the titles and abstracts of all identified articles and legal acts to ascertain their eligibility according to the inclusion criteria. Subsequently, the full-text articles and relevant legal acts were meticulously reviewed by three authors (AZ, AT, and AG) for potential inclusion. In cases where differences of opinion arose, they were resolved through deliberative discussions led by a fourth author (GK).

Data synthesis in this study was conducted following the Arksey and O’Malley framework, utilizing a descriptive approach to concisely summarize the primary findings and identify common themes and patterns among the selected studies (24). The evaluation of the included studies and legal acts was based on their relevance to the research question and their legal significance, thereby assessing their quality.

## 3. Assessment of policy

This review highlights the limited research conducted on support measures for caregivers in Kazakhstan, as evidenced by the scarcity of peer-reviewed articles on this topic. To bridge this research gap, we have explored the “grey literature” to gather relevant information. Our analysis entails summarizing the key laws, standards, and regulations pertaining to the medical and social challenges associated with informal caregiving for the older adults. Furthermore, we have examined the availability of support for informal caregivers of the older adults within the legal framework of the Republic of Kazakhstan. Additionally, we have scrutinized the legal aspects concerning the support provided to employees who care for their family members, including provisions for reduced working hours, paid or unpaid leave, and financial compensation. Through our search process, we have identified the primary legal acts addressing the medical and social issues concerning caregivers in Kazakhstan. The key provisions are presented in Supplementary Table S3. Moreover, we have analyzed and ranked the state policies regarding caregiver support in our country, making comparisons with international experiences.

### 3.1. Introduction of the term caregiver

Older adults individuals who face functional impairment, disability, or chronic illness rely on assistance to compensate for their reduced ability to carry out daily activities. This support can be obtained through both formal and informal care. Formal care is administered by governmental organizations, local, national, or international non-governmental organizations (NGOs), or commercial entities. It often involves professional caregivers such as nurses, doctors, social workers, and hired nurses. On the contrary, informal care entails the support provided by family members, neighbors, friends, and volunteers.

Until recently, the term “caregiver” was absent from the legislative documents of the Republic of Kazakhstan. However, with the recent addition of the Law of the Republic of Kazakhstan “On Special State Benefits” in 2022, the concept of a “caregiver” was introduced for the first time. According to this law, a caregiver is defined as an individual who directly provides care to a person with a first-degree disability, irrespective of their familial relationship. The status of a caregiver is granted to only one individual upon the request of a person with a first-degree disability, and it is based on a disability certificate (25).

According to the legislation of the Republic of Kazakhstan, individuals are classified as having a first-degree disability if they exhibit persistent, significant, or pronounced impairments of bodily functions resulting from diseases, consequences of injuries, or defects. These impairments lead to a pronounced limitation in their ability to perform one or more categories of life activities (26).

In the classification system, limitations in life activities are categorized into three degrees based on their severity. The first degree signifies a partial limitation in performing life activities. The second degree indicates that an individual is capable of carrying out life activities partially or with the assistance of unauthorized individuals. The third degree pertains to individuals who are completely dependent on others to carry out life activities.

To determine a first-degree disability, the criteria for life activities should be assessed at the third degree for one or several the following indicators: self-service, mobility, work capacity (labor activity), ability to learn, orientation (or inability to orientate, or disorientation), communication, and self-control.

### 3.2. Legislative norms in older adults care provision

Article 27 of the Constitution of the Republic of Kazakhstan emphasizes the significance of family values and social welfare, explicitly stipulating the duty of able-bodied children to assume the responsibility of caring for their disabled older adults parents and grandparents (27). Additionally, Article 145 of the Code of Marriage and Family elaborates on the legal obligations imposed on children to fulfill their caregiving duties. Failure to meet these obligations can result in various repercussions, as outlined in in Article 146 of the Code on Marriage and Family. These consequences may include the requirement for children to contribute to alimony payments and cover additional expenses arising from parental illness or the need for external caregiving services (28).

### 3.3. Social welfare

Social support in the Republic of Kazakhstan operates within the legal framework established by the Social Code. As per this Code, special social services are defined as a comprehensive range of services designed to address objective barriers that impede individuals or families from leading fulfilling lives. The primary objective of these services is to promote equal opportunities for community integration, fostering social cohesion among citizens (29). The legislation governs the provision of specialized social services, specifically targeting individuals or families facing challenging life circumstances. These circumstances may arise from factors such as illness, injury, age, or disability, resulting in a complete or partial loss of self-care ability, mobility, or access to necessities. The Code encompasses a range of services, including social and household support, socio-medical assistance, socio-psychological counseling, socio-pedagogical interventions, social and occupational guidance, socio-cultural activities, socio-economic aid, and socio-legal assistance (29).

However, delivering the full range of services necessitates a multidisciplinary team comprising not only healthcare and social welfare professionals but also legal experts, economists, and psychologists. This is due to the complex nature of the required assistance, which extends beyond the scope of a social worker’s capabilities alone. Persistent challenges exist, including low qualifications of social workers, substandard quality of care and support services provided to those in need, inadequate development of home assistance programs, and a shortage of trained social workers Currently, a mere 1.3% of the older adults population in the Republic of Kazakhstan receives social services and home care, underscoring the limited reach of these services. The home-based services primarily encompass household cleaning, grocery shopping, and medication procurement, with the associated expenses borne by the clients themselves (30). Notably, that older adults individual facing difficult life circumstances, who have able-bodied adult children or a spouse, are ineligible for special social services at home (31).

In addition to the provision of home care services, the Republic of Kazakhstan offers specialized social services in the realm of social welfare for the population in hospitals and semi-hospital settings. These services are designed for individuals who require long-term or temporary (up to 6 months) daytime stay and, and their funding is provided by the government (31, 32). However, it is noteworthy that Kazakhstan has a limited number of institutions, amounting to only 13, that provide palliative and medical care, encompassing hospices, nursing centers, and departments for symptomatic treatment and palliative care. The collective bed capacity of these facilities does not exceed 500, potentially indicating inadequate resources to accommodate all patients in need. Furthermore, individuals residing in remote areas often face challenges in accessing palliative and nursing care, thereby exacerbating the barriers to care for those who require it (6, 33). The development of alternative palliative care options such as mobile teams, home hospices, and day care centers, remains relatively insufficient (6). This situation can be attributed, in part to the relatively lower allocation of state budget expenditures in the Republic, which range from 3.5 to 4.5 times lower in the social sphere compared to more socially and economically developed countries. In situations where additional private care services are necessary, the recipients of social services bear the responsibility of covering the associated costs (31).

In accordance with the key documents concerning social support, caregiver support involves providing essential training to family members regarding the fundamental aspects of home healthcare. Furthermore, health and social service professionals have the responsibility of delivering social and psychological assistance to family members who reside with individuals benefiting from these services. The ultimate objective is to create a nurturing psychological environment while effectively addressing and resolving conflicts (29, 34).

### 3.4. Work-care balance

#### 3.4.1. Part-time work

Balancing work responsibilities with caregiving duties for older adults individuals frequently presents challenges. Caregivers may encounter difficulties in maintaining their financial income, which can sometimes result in a complete loss of income. Addressing this issue, Article 70 of the Labor Code of the Republic of Kazakhstan include provisions for part-time employment for employees caring for sick family members. Notably, reduction in working hours does not impede the employee’s entitlement to paid annual leave, calculation of work experience, or other labor rights (35).

#### 3.4.2. Older adults care leave

The Labor Code of the Republic of Kazakhstan includes provisions for paid parental leave (35). However, it does not extend the same support to individuals taking leave to care for older adults relatives. In contrast, France has implemented a family leave policy that considers regular caregiving for older adults relatives on par which childcare (36). In Kazakhstan, similar to Russia, employees have the option to take unpaid leave for family and other valid reasons, with the duration determined through agreement between the employee and employer (35, 37, 38).

It is crucial to highlight that caregivers for the older adults in Kazakhstan are ineligible to obtain sick leave on behalf of their older adults relatives (39). This particular circumstance can present supplementary difficulties for individuals who are obliged to fulfill caregiving responsibilities for their older adults relatives.

### 3.5. Caregiver allowance

In the Republic of Kazakhstan, caregiver allowances are exclusively provided to individuals who care to those classified as having a first-degree disability (40). The enactment of the Social Code (41) has resulted in an increase in the amount of the caregiving allowance, now set at 1.61 times the subsistence minimum, compared to 1.4 times the subsistence minimum in 2021 (40). The subsistence minimum refers to the minimum cash income per person, reflecting the cost of a basic food basket.

According to the Law on Minimum Social Standards and their Guarantees, the subsistence minimum denotes the minimal monetary income per individual, equivalent in value to the expenses associated with the basic food basket. The basic food basket represents a basic assortment of essential food items, commodities, and services required to sustain human life, both in tangible and monetary terms. It comprises of: (a) the food basket; and (b) a fixed portion of non-food items and services. The determined value for the minimum subsistence amount, used for calculating the extent of social payments for the year 2023, stands at 40,567 tenge (42).

### 3.6. Healthcare support

In Kazakhstan, the provision of medical services operates through a dual system consisting of both public and private sectors. The public healthcare system functions under the framework of compulsory health insurance, where in both employers and employees contribute to the health insurance system to a certain extent. As per the legislation of the Republic of Kazakhstan, individuals who are not employed but are responsible for caring for a person classified as having first-degree disability have their medical service contributions paid by the state (43). However, individuals caring for the older adults who do not meet the criteria for disability classification do not have access to planned public health services. In such instances, caregivers are required to make private health insurance payments, which can pose challenges if they encounter unemployment and subsequent loss of income.

## 4. Actionable recommendation

Policy changes

- A crucial step towards recognizing and supporting caregivers who are not affiliated with formal medical or social organizations is to develop and incorporate the concept of “informal (family) care” into legislation. By introducing this concept into the legislation, Kazakhstan can acknowledge the valuable role played by informal caregivers and establish a legal framework that safeguards their rights and provides the necessary support.
- It is essential to develop and introduce the term “caregiver” into the legislation, which should extend beyond individuals caring for first-degree disabled individuals. This development will help determine the specific individuals who qualify as caregivers.
- It is necessary to develop comprehensive criteria for assessing the caregiving needs of individuals aged 65 and older. Based on these assessments, various levels of medical and social support should be formulated. This approach ensures that older adults individuals and their caregivers receive the appropriate range of medical and social services according to the severity of their condition.
- It is crucial to establish clear criteria for transitioning to part-time work and caregiving leave. Additionally, it is necessary to include caregivers who provide temporary supervision for older adults individuals when they fall ill in the list of individuals eligible for sick leave. This inclusion will grant caregivers the required flexibility in their employment while fulfilling their caregiving responsibilities. Prioritizing the establishment of these criteria is imperative for effectively supporting caregivers.

Raising public awareness and changing societal attitudes toward caregiving

- Raise awareness about the indispensable role of caregivers and the challenges they encounter to foster understanding, empathy, and recognition within society.
- Increase the involvement of volunteers in the provision of social services, enhance accountability, and engage capable family members in supporting and caring for older adults individuals.
- Collaborate with media outlets to promote positive depictions of caregivers and share their stories, highlighting their contributions and inspiring others to support and appreciate caregivers.

Strengthening collaboration between healthcare and social service providers

- Establish interdepartmental collaboration between medical and social services, including the consolidation of client databases and the creation of a legal framework to facilitate this cooperation.
- Develop joint training programs that bring together healthcare professionals and social service providers, enhancing their understanding of caregivers’ needs and promoting effective teamwork.
- Implement integrated care plans involving both healthcare and social service providers, facilitating joint assessment, planning, and the delivery of comprehensive care for older adults individuals.

Development and implementation of a comprehensive caregiver support program

- Provide specialized training programs for caregivers, focusing on topics such as caregiving techniques, self-care, and managing the needs of older adults individuals. This training will equip caregivers with the necessary knowledge and skills to effectively care for both themselves and the older adults, ultimately reducing stress and anxiety.
- Establish support groups and counseling services to offer emotional support and guidance to caregivers, allowing them to share their experiences and seek advice from professionals. Introduce respite care services that temporarily relieve caregivers from their responsibilities, enabling them to take breaks and attend to their own needs.
- Providing financial assistance is essential to alleviate the financial burden experienced by caregivers. This support can be facilitated through the utilization of legal and economic mechanisms such as care allowances, tax deductions, health insurance, and pension contributions. Financial support becomes particularly crucial when caregivers are compelled to forego employment in order to fulfill their caregiving responsibilities.
- It is imperative to develop a comprehensive process for the identification, screening, assessment of the caregiving burden and quality of life, and provision of specialized medical and psychological assistance to caregivers, while simultaneously guaranteeing their access to essential support and resources.

## 5. Discussion

We conducted a scoping review and synthesis of policies and practices related to informal care in the Republic of Kazakhstan. Throughout our review, we identified six main themes, which include the introduction of the term “caregiver,” legislative norms in older adults care provision, social welfare, work-care balance, caregiver allowance, and healthcare support.

Revision of legislation to incorporate the concept of “informal (family) care” is of utmost importance, along with the development and implementation of legal norms that regulate informal (family) care in the Republic of Kazakhstan, drawing upon the best global practices. The most commonly used definition of an informal caregiver in the literature is as follows: “An informal caregiver refers to a non-professional individual who voluntarily provides care or assistance, at any given time, to a family member, friend, neighbor, or any other person with a long-term mental or physical illness, disability, or age-related condition” (44). As per the German Social Code, informal caregivers are defined as “individuals who provide non-professional care to individuals who are unable to care for themselves due to health issues.” Additionally, the requirement for care must be continuous for a minimum duration of 6 months and reach a certain level of severity (45).

In Portugal, Decree 2022 outlines the criteria for recognizing informal caregivers and establishes support measures for them. The status of an informal caregiver can only be granted to one applicant per household, provided that they reside with the care recipient and offer full-time care. Moreover, the caregiver must not be engaged in any paid professional or other activities that would conflict with their responsibility of providing continuous care to the individual in need (46, 47).

The legal obligation of providing care for older adults family members being the responsibility of their children is a widely acknowledged norm in numerous countries, such as China, Bangladesh, India, Singapore, Brazil, Mexico, Russia, Turkey, Algeria, Argentina, Chile, Singapore (48, 49). This norm is also observed in the Republic of Kazakhstan. In Japan, under the previous civil code, the eldest son was entitled to inherit the family’s property, and his wife, as the daughter-in-law, bore a legal and moral responsibility to care for her husband’s parents. However, with the introduction of the New Civil Code in 1947, the responsibility for parental care was extended equally to all children. This legal provision remained the societal norm until recently, when the National Long-term Care Insurance Act was enacted. This act establishes a comprehensive set of measures for older adults care, funded through insurance premiums (50).

However, in Scandinavian countries such as Sweden, Denmark, Netherlands, and Norway, long-term care has been recognized as a social risk factor since 1980. As a result, the primary legal responsibility for older adults care has been assigned to the state. In these countries, the contribution of the family to informal care provision is relatively lower compared to countries where family care is the predominant approach. On the other hand, countries like Austria, Belgium, Canada, Germany, France, Switzerland, the United Kingdom, and the United States share the responsibility for care between the state and the family. Their systems combine government-provided universal benefits and means-tested caregiver’s allowance (51).

In addition to legislation, the cultural and social characteristics of each country play a significant role in shaping the perception of older adults care. In Asian culture traditional values and cultural norms continue to emphasize the sons’ responsibility for older adults care alongside state policies (52–56). Similarly, in Kazakhstan, national family traditions still serve as an important source of support for the older adults (5). However, there has been a recent shift towards a change in family composition, with a transition from extended families to nuclear families and the older adults living separately from their children. This change may lead to an increased demand for formal care services.

In Kazakhstan, to be eligible for special social services, certain criteria must be met, including reaching retirement age and living alone within a community or being disabled (29). However, in countries such as the United States, Germany, and Japan, publicly funded long-term care services are established based on a comprehensive care needs assessment, which plays a crucial role in determining the provision of social services (57, 58). The competency framework considers functional disability, which is assessed based on the individual’s ability to perform activities of daily living (ADL), instrumental activities of daily living (IADL), and cognitive tasks. In Germany, for instance, there are five levels of care needs depending on the degree of functional disability, each corresponding to a different package of medical and social assistance (59).

It is important to highlight that the right to part-time employment for informal caregivers in Kazakhstan applies exclusively to family members and relatives. Consequently, if the caregiver is assisting a distant relative, friend, or neighbor, transitioning to part-time work may present certain difficulties. Similar situations are observed in countries such as Germany (60), Japan (61), Great Britain (62), and France (36), where the scope of informal care recognized by employers is limited to family members and relatives. Nonetheless, unlike the legislation in the aforementioned countries, the Labor Code of Kazakhstan does not specify the conditions for part-time employment. It does not provide information regarding the length of employment required at a specific enterprise or company before exercising this right, the duration of the permitted part-time arrangement, or the types of enterprises and job positions eligible for this right. Furthermore, the Labor Code does not mention the possibility of transitioning to remote work if deemed necessary.

In Kazakhstan, unlike many countries where implemented leave systems specifically designed to cater to the long-term care of close relatives, such provisions are not currently in place. Generally, the policies regarding payment for sick leave differ from those for caregiving leave, with sick leave typically providing a limited number of days with full wage restoration. For instance, several states in the United States, including California, Connecticut, Massachusetts, Oregon, and Vermont, have implemented paid sick leave laws that allow workers to use sick leave when caring for sick family members (63).

While some states offer unpaid leave, others provide compensation to address caregiving needs. For instance, Austria, the Czech Republic, Luxembourg, and Germany have established paid leave for older adults care (46). In Germany, workers can avail themselves of a short-term leave program for up to 10 days when nursing care is required, with 90% of their wages covered through a caregiver allowance (64). In Canada, individuals caring for terminally ill close relatives are entitled to up to 28 weeks of unpaid family leave per year (65). The US Family and Medical Leave Act (FMLA) allows for up to 12 weeks of unpaid leave within a 12-month period to address short- or long-term caregiving needs (66). Some states, like California and New York, have introduced paid family leave at the state level (67), wherein workers receive 67% of their wages during care leave (68). In Japan, a system exists where two-thirds of the salary is compensated for a certain period through unemployment insurance for individuals on care leave (61). In France, workers have the right to up to three months of unpaid care leave, which can be renewed within one year (36).

Paid leave is considered one of the most effective and practical ways to support long-term care workers. Having access to paid care leave offered by employers significantly facilitates the ability to provide regular or temporary informal care to older adults parents and relatives (69). Unfortunately, in countries where such policies are lacking, caregivers may face challenges in balancing their caregiving responsibilities with their work obligations.

In Kazakhstan, benefits are primarily designated for the support and assistance of individuals classified as belonging to the first-degree disability. However, it is noteworthy that over two-thirds of the member countries in the Organization for Economic Co-operation and Development (OECD) have implemented schemes to provide financial compensation to informal caregivers. These care allowances are distributed either directly to caregivers in the form of a caregiver allowance or as compensation to care recipients. Several nations, such as Netherlands, Sweden, the United Kingdom, and Germany, have adopted a dual approach, offering both types of care allowances to aid their citizens in the provision of care for their loved ones (46).

In Germany, caregivers are eligible to receive a caregiver allowance if they provide regular care for a minimum of 10 hours per week to one or more individuals requiring assistance (45). In Canada, caregivers are provided with a caregiver allowance amounting to 55% of the average weekly wage for the duration of their leave. It should be noted that individuals receiving this benefit are not permitted to engage in full-time employment during this period (65).

In Russia, the allowance for older adults care is provided to individuals who care for those over the age of 80, regardless of their family relationship or cohabitation. The allowance, amounting to 1,200 rubles, is credited monthly to the recipient’s pension account. The caregiver must be unemployed but capable of working according to pension legislation and should not be receiving unemployment benefits from the employment service (70).

In the United States, Medicaid offers care allowances to adult children, relatives, or grandchildren who provide care. The caregiver is expected to receive compensation ranging from 1,550 to 2,550 dollars per month, depending on the level of care required by their aging parent and their state of residence (71).

The current healthcare system in the Republic of Kazakhstan lacks consideration for the challenges and requirements of individuals providing home care for the older adults. There is a lack of established legal framework addressing the identification, assessment, and provision of specialized medical and psychological assistance for these caregivers.

Within the strategic documents of the Ministry of Health of the Republic of Kazakhstan, which govern the provision of medical and psychological assistance, the term “guardian” is solely acknowledged in relation to individuals responsible for caring for individuals classified under the first-degree disability. Consequently, caregivers receive healthcare services as regular patients, without consideration for their specific needs. Family members who are providing care for an older adults individual are perceived merely as individuals offering assistance to their older adults relative (72).

In Germany, if a caregiver decides to cease employment in order to provide care, their health insurance benefits will continue throughout the duration of their caregiving responsibilities, as long as they were enrolled in the insurance system prior to assuming their caregiving duties (73).

In the United States, the CARE Act (74) mandates that healthcare providers recognize and register family caregivers, inform them about procedures performed on the older adults, and provide instructions on the tasks expected of caregivers. In Portugal, the assessment of the quality of life and the burden experienced by informal caregivers is legally mandated. Health and social development services also aim to provide psychosocial support to informal caregivers. The importance of rest for informal caregivers is also emphasized. Health services have established self-help groups facilitated by healthcare professionals to foster mutual support and the sharing of experiences among individuals who have lived or are currently experiencing similar situations and challenges, thus minimizing potential feelings of isolation (47).

## 6. Conclusion

Ensuring high-quality medical and social care should be the primary focus in the development of legal regulations and state policies in the medical and social sectors in the Republic of Kazakhstan. The current system of medical and social support for informal caregivers of the older adults in Kazakhstan requires significant reforms. It is crucial to revise the legislation to incorporate the concept of “informal (family) care” and to develop and implement legal norms that regulate informal (family) caregiving. Additionally, establishing a robust legal framework that governs informal (family) caregiving relationships is essential for both caregivers and employers to establish employment relationships that consider the needs of long-term caregivers. Moreover, implementing a caregiver registration system and conducting screenings to assess care-related burden, as well as the physical and mental health and care-related needs are important for effective monitoring.

## Author contributions

AZ, GK, and AG contributed to conception and design of the study. AZ wrote the first draft of the manuscript. AZ, GK, and AT wrote sections of the manuscript. All authors contributed to the article and approved the submitted version.

## Conflict of interest

The authors declare that the research was conducted in the absence of any commercial or financial relationships that could be construed as a potential conflict of interest.

## Publisher’s note

All claims expressed in this article are solely those of the authors and do not necessarily represent those of their affiliated organizations, or those of the publisher, the editors and the reviewers. Any product that may be evaluated in this article, or claim that may be made by its manufacturer, is not guaranteed or endorsed by the publisher.
