# Supplementary material for: Measures to support informal care for the older adults in Kazakhstan: a review of the current status
Source: Front Public Health. 2023 Aug 21;11:1247684. doi: 10.3389/fpubh.2023.1247684 (PMC10475596; doi:10.3389/fpubh.2023.1247684)
Supplement: Supplementary file 1 [file Table_1.DOCX]

Supplementary Material

Measures to support informal care for the elderly in Kazakhstan: A Review of the Current Status

Aliya Zhylkybekova^1^*, Andrey Turlayev^2^, Andrej M. Grjibovski^,3,4,5,6^, Gulbakit K. Koshmaganbetova^1^

*** Correspondence: Aliya Zhylkybekova,** [**zhylkybekovaa@gmail.com**](mailto:zhylkybekovaa@gmail.com)

**Supplementary Table 1.** List of Websites of government ministries of the Republic of Kazakhstan

| Websites | Links |
| --- | --- |
| Adilet Legal Information System of Regulatory Legal Acts | <https://adilet.zan.kz/eng> |
| Ministry of Labor and Social Protection of the Population of the Republic of Kazakhstan | <https://www.gov.kz/memleket/entities/enbek?lang=en> |
| Ministry of Healthcare of the Republic of Kazakhstan | <https://www.gov.kz/memleket/entities/dsm/about?lang=en> |
| e-Government of the Republic of Kazakhstan. | <https://egov.kz/cms/en> |

**Supplementary Table 2**. Search strategy

| **Data Base** | **Search strategy** |
| --- | --- |
| PubMed | ((((((((Caregivers) OR ("family caregivers")) OR ("informal caregivers")) AND (elderly)) OR ("old-aged")) OR "functionally impaired elderly" AND ("caregiver support")) OR ("social support")) OR "psychosocial Support Systems" OR ("health policy")) AND (Kazakhstan) AND ((y_5[Filter]) AND (ffrft[Filter]) AND (fha[Filter])) AND ((y_5[Filter]) AND (ffrft[Filter]) AND (fha[Filter])) |
| Web of Sciences | (((((ALL=(caregivers[TS] OR caregivers [AB] OR "family caregivers [TS] OR caregivers [AB] OR caregivers [AK] AND "caregiver support"[AB] OR"social support" [AB] OR "health policy"[AB] AND Kazakhstan [TS] OR Kazakhstan [AB] OR Kazakhstan [AK] )) AND ALL=((((((ALL=(caregivers[TS] OR caregivers [AB] OR "family caregivers" [TS] OR caregivers [AB] OR caregivers [AK] AND "caregiver support"[AB] OR "social support" [AB] OR "health policy"[AB] AND Kazakhstan [TS] OR Kazakhstan [AB] AND Kazakhstan [AK] )) AND ALL=(caregivers[TS] OR caregivers [AB] OR "family caregivers" [TS] OR caregivers [AB] OR caregivers [AK] AND "caregiver support" [AB] OR «social support" [AB] OR "health policy"[AB] AND Kazakhstan [TS] OR Kazakhstan [AB] ORKazakhstan [AK] )) ))) ) and Open Access and 1.112 Palliative Care or 1.14 Nursing (Citation Topics Meso) and 1.112.237 Palliative Care or 1.112.161 Dementia Caregivers or 1.112.297 Cancer Survivors (Citation Topics) and 2023 or 2022 or 2021 or 2020 or 2019 or 2018 (Publication Years) and Article or Early Access or Review Article (Document Types) and Public Environmental Occupational Health or Health Care Sciences Services or Gerontology (Web of Science Categories)297 Cancer Survivors (Citation Microtopics) and 2023 or 2022 or 2021 or 2020 or 2019 or 2018 (Publication Years) and Article or Early Access or Review Article (Document Types) and Public Environmental Occupational Health or Health Care Sciences Services or Gerontology (Web Categories of Science)297 Cancer |
| Scopus | TITLE-ABS-KEY ( caregivers ) OR "family caregivers" OR "informal caregivers" AND elderly OR "old-aged" OR "functionally impaired elderly" AND "caregiver support" OR "social support" OR "psychosocial Support Systems" OR "health policy" AND kazakhstan AND PUBYEAR > 2017 AND PUBYEAR < 2024 AND ( LIMIT-TO ( AFFILCOUNTRY , "Kazakhstan" ) ) |

**Supplementary Table 3**. Legislation Supporting Caregivers in Kazakhstan, 1995-2023

| Name of law | Year  enacted | Context of law | Main requirements |
| --- | --- | --- | --- |
| Constitution of the Republic of Kazakhstan | 1995 | Article 27. “Overage able-bodied children are obliged to take care of disabled parents.” | It is a constitutional obligation for everyone to take care of their disabled parents in various ways, including providing informal care. |
| Code "On marriage (matrimony) and family" | 1995 | Article 145. “Overage able-bodied children are obliged to support and take care of their non-self-sufficient parents in need of assistance.” | It is the responsibility of each person to support and care for their disabled parents who need help in various ways, including the implementation of informal care for them. |
|  |  | Article 146. “In the absence of care from adult children for their disabled parents and in exceptional circumstances, the court may compel adult children to contribute to the additional expenses incurred as a result of these circumstances” |  |
| Code "On the health of the people and the healthcare system" | 2020 | Article 126. “Palliative care is a set of services aimed at improving the quality of life of patients with severe and incurable diseases (conditions), as well as their families and caregivers. Palliative care includes medical, social services and spiritual support.” | This article divides palliative care into medical palliative care and palliative care provided by others, including family members, who can provide medical, social, and spiritual support. |

| Social Code | 2023 | Article 163. “Individual assistant services are provided for persons with disabilities in accordance with the individual program”  “The services of an individual assistant for a person with a first – degree disability, who has difficulty in moving are not provided when assigning and paying state benefits to a person caring for him” | Persons with disabilities are entitled to a personal assistant or form a caregiver at will. |
| --- | --- | --- | --- |
|  |  | Article 168. “Persons with disabilities carry out orders for the purchase of goods and (or) services in accordance with the recommendations in the individual program.” | Persons with disabilities have access to necessary goods and services provided by the state |
|  |  | Article 188. “A caregiver's allowance shall be paid monthly at the expense of budget funds in the amount of 1.61 subsistence minimum.” | Caregivers of individuals classified under the first – degree disability are eligible to receive a caregiver allowance. |
|  |  | Article 206. “The period of participation in the pension system in the provision of the state basic pension payment shall include the time of caring for a person with a first – degree disability, a single person with a second – degree disability and an old-age pensioner in need of assistance, as well as for an elderly person who has reached the age of eighty” | The obligation of the state to pay contributions to the Pension Fund for a person who is not in an employment relationship and cares for a person with a first – degree disability. |

|  |  | Article 208. “Calculating the length of service for assigning pension payments by age, the following are taken into account: the time of caring for a person with a first – degree disability, a single person with a second – degree disability and an old-age pensioner in need of assistance, as well as an elderly person who has reached the age of eighty” | When recalculating pension accruals based on age, a caregiver has the potential to accumulate additional years of credited service for the time spent providing care |
| --- | --- | --- | --- |
| Labor Code of the Republic of Kazakhstan | 2016 | Article 70. ”Upon a written application from an employee, who is caring for a sick family member in accordance with a medical report, the employer is obligated to establish a part-time work regime for the employee” | Possibility of establishing part-time work in case of need to care for a sick family member |
|  |  | “Part-time work does not entail restrictions for the employee on the duration of paid annual leave, calculation of work experience and other rights in the field of labor established by this Code, labor, collective agreements, agreements” | Does not affect the duration of paid annual leave, the calculation of seniority and other labor rights |
| Geriatric care standard | 2021 | Chapter 3. “consulting patients and their relatives on treatment and rehabilitation, determining the scope and rational methods of examination in order to obtain the most complete and reliable diagnostic information, including at home for patients with a sharply reduced or lost ability to self-service.  “determining the degree of loss of the patient’s ability to self-service and compiling a medical and social prognosis using questionnaires, as well as the Barthel scale” | Counseling relatives on treatment and rehabilitation  The assessment of the functional capacity of elderly individuals is conducted using the Barthel scale. |
| Law “On amendments and additions to certain legislative acts of the Republic of Kazakhstan on issues of social protection of certain categories of citizens” | 2021 | Article 1 is supplemented by subparagraph 1-1) of the following content:  “caregiver is an individual who directly cares for a person with a first - degree disability, regardless of family ties with him” | Added definition of “caregiver”, only for caregivers who care for person with a first - degree disability |
| The Law of the Republic of Kazakhstan On Special Social Services | 2017 | Article 6. The criteria by which an individual or a family can be acknowledged as being in a challenging life circumstance include "inability to perform self-care tasks due to advanced age, illness, and/or disability. | The law provides for the provision of special social services to elderly people in difficult life situations. |
| Order of the Minister of Health of the Republic of Kazakhstan “On approval of the standard for organizing the provision of medical and social assistance in the field of mental health to the population of the Republic of Kazakhstan” | 2021 | “consulting patients and their relatives on treatment and rehabilitation, determining the scope and rational methods of examination in order to obtain the most complete and reliable diagnostic information, including at home for patients with a sharply reduced or lost ability to self - service | The right to provide consultative and diagnostic assistance to caregivers.  The right to conduct psychoprophylactic consultations |
| Order of the Minister of Health of the Republic of Kazakhstan “On approval of the Standard for organizing the provision of primary health care in the Republic of Kazakhstan” | 2016 | “A social worker, when providing special social and medical services, performs the following actions: provision of special social services aimed at maintaining and improving the health of service recipients, including: teaching family members the basics of home health care” | Family members have right for consultation the basics of home health care. |
| Order of the Minister of Health of the Republic of Kazakhstan On amendments and additions to the order "On approval of the rules for keeping records of consumers of medical services and granting the right to receive medical care in the system of compulsory social health insurance” | 2020 | Chapter 2. The procedure for keeping records of consumers belonging to the category of persons whose contributions are paid by the state according to the following priority categories:  7) “a non-working person caring for a person with a first - degree disability” | The obligation of the state to pay contributions to the Social Health Insurance Fund for a person who is not in an employment relationship and cares for a person with a first – degree disability. |

We have made efforts to include all available links related to social and medical support for informal care in Kazakhstan. However, it is important to acknowledge that certain publications available in the republican institutional journals, which contain information about the existing model and challenges of social support for long-term care for the elderly, may not have been included in this review. The reason for this omission is that these articles are published in Russian, and thus, they may not be informative for readers who do not understand the language. We will provide links to publications in our response, and if necessary, we will include them in the Supplementary materials (1-5).

1. Akanov A, Tulebaev K, Eshmanova A, CHajkovskaya V, Abikulova A, Kalmahanov S. Analiz sostoyaniya i perspektivy razvitiya geriartricheskoj pomoshchi naseleniyu Kazahstana. Uspekhi gerontologii. 2014;27:89-95.

2. Pritvorova T, Bektleeva D. Upravlenie sistemoj social'nyh uslug v sovremennoj ekonomike. Sbornik konferencij NIC Sociosfera. 2013:59-67.

3. Pritvorova T, Bektleeva D. Upravlenie sistemoj dlitel'nogo uhoda za pozhilymi lyud'mi v Kazahstane v kontekste effektivnyh praktik razvityh stran. Vestnik Omskogo universiteta. 2014.

4. Sidorenko A. Aktivnoe, zdorovoe i dostojnoe dolgoletie dlya Kazahstana. Almaty; 2020.

5. Sidorenko AV, Eshmanova AK, Abikulova AK. [Population aging in Kazakhstan. 1. Problems and opportunities]. Adv Gerontol. 2017;30:505-15.
